# Responses of four submerged macrophytes to freshwater snail density (*Radix swinhoei*) under clear‐water conditions: A mesocosm study

**DOI:** 10.1002/ece3.6489

**Published:** 2020-06-23

**Authors:** Yongwei Zhi, Yang Liu, Wei Li, Yu Cao

**Affiliations:** ^1^ Key Laboratory of Aquatic Botany and Watershed Ecology Wuhan Botanical Garden Chinese Academy of Sciences Wuhan China; ^2^ Hubei Key Laboratory of Wetland Evolution & Ecological Restoration Wuhan Botanical Garden Chinese Academy of Sciences Wuhan China; ^3^ University of Chinese Academy of Sciences Beijing China

**Keywords:** antiherbivory, clear water, negative effects, snail density, species‐specific, submerged macrophytes

## Abstract

Macrophytes play a key role in stabilizing clear‐water conditions in shallow freshwater ecosystems. Their populations are maintained by a balance between plant grazing and plant growth. As a freshwater snail commonly found in shallow lakes, *Radix swinhoei* can affect the growth of submerged macrophytes by removing epiphyton from the surface of aquatic plants and by grazing directly on macrophyte organs. Thus, we conducted a long‐term (11‐month) experiment to explore the effects of snail density on macrophytes with distinctive structures in an outdoor clear‐water mesocosm system (with relatively low total nitrogen (TN, 0.66 ± 0.27 mg/L) and total phosphorus (TP, 36 ± 20 μg/L) and a phytoplankton chlorophyll *a* (Chl*a*) range of 14.8 ± 4.9 μg/L) based on two different snail densities (low and high) and four macrophyte species treatments (*Myriophyllum spicatum*, *Potamogeton wrightii*, *P. crispus*, and *P. oxyphyllus*). In the high‐density treatment, snail biomass and abundance (36.5 ± 16.5 g/m^2^ and 169 ± 92 ind/m^2^, respectively) were approximately twice that observed in the low‐density treatment, resulting in lower total and aboveground biomass and ramet number in the macrophytes. In addition, plant height and plant volume inhabited (PVI) showed species‐specific responses to snail densities, that is, the height of *P. oxyphyllus* and PVI of *M. spicatum* were both higher under low‐density treatment. Thus, compared with low‐density treatment, the inhibitory effects of long‐term high snail density on macrophytes by direct feeding may be greater than the positive effects resulting from epiphyton clearance when under clear‐water conditions with low epiphyton biomass. Thus, under clear‐water conditions, the growth and community composition of submerged macrophytes could be potentially modified by the manual addition of invertebrates (i.e., snails) to lakes if the inhibitory effects from predatory fish are minor.

## Graphical abstract

In the study, we conducted a long‐term (11‐month) experiment to explore the effects of snail density on macrophytes with distinct structures in an outdoor clear‐water mesocosm based on different snail density and macrophyte species treatments. Our results indicated that high snail density reduced total biomass of submerged macrophytes and ramet number. In addition, plant height and plant volume inhabited showed species‐specific responses to snail densities.

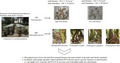

## 1 INTRODUCTION

Submerged macrophytes play important roles in freshwater ecosystems by contributing to the establishment and maintenance of clear water (Jeppesen, Lauridsen, Kairesalo, & Perrow, 1998; Scheffer, Hosper, Meijer, Moss, & Jeppesen, 1993; Schriver, Bøgestrand, Jeppesen, & Søndergaard, 1995; Zhi, Cao, Sun, Li, & Jeppesen, 2018). For example, submerged macrophytes can reduce sediment resuspension and internal nutrient loading (Carpenter & Lodge, 1986; Jeppesen et al., 1998). Macrophyte growth can be influenced by both biotic and abiotic factors, including light and nutrition (Carpenter & Lodge, 1986; Yuan, Li, Liu, & Deng, 2012). Epiphyton can negatively influence macrophyte growth by reducing light conditions and nutrient availability for host plants, especially in eutrophic lakes (Cao et al., 2017; Jones et al., 1999; Phillips, Eminson, & Moss, 1978). Moreover, increased epiphyton biomass is correlated with higher leaf complexity in macrophytes, as indicated by fractal dimension studies (Ferreiro, Giorgi, & Feijoó, 2013; Hao et al., 2017). For example, the fractal dimensions of *Potamogeton lucens*, *P. crispus*, and *Myriophyllum spicatum* leaves (1.12, 1.32, and 1.68, respectively) suggest that higher plant leaf complexity results in larger leaf fractal dimensions (Dibble & Thomaz, 2009; Hao et al., 2017). Thus, the heterogeneity of macrophytes with distinct structures can affect epiphyton and therefore food availability of the macroinvertebrate community (Ferreiro, Feijoó, Giorgi, & Leggieri, 2010).

Snails are large invertebrates commonly found in lake systems and mainly feed on periphytic algae, bacteria, detritus complexes, and decaying macrophytes (Brönmark, 1990; Lodge, Cronin, Donk, & Froelich, 1998). Previous studies have shown that a complex relationship exists among snails, epiphyton, and macrophytes (Brönmark, 1989; Jones et al., 1999; Thomas, 1990; Underwood, Thomas, & Baker, 1992). For example, snails can promote submerged macrophyte growth by removing epiphyton from the surface of aquatic plants and reducing the impact of shading and nutrient competition (Cao, Li, & Jeppesen, 2014; Jones & Sayer, 2003; Underwood et al., 1992). However, snails can also inhibit macrophyte growth by direct grazing behavior (Elger & Lemoine, 2005; Xiong, Yu, Wang, Liu, & Wang, 2008), especially under high snail density (Li, Liu, & Gu, 2009; Sheldon, 1987). Brönmark (1990) stated that the gut contents of herbivorous snails in their natural state are dominated by algae and organic detritus, with vascular plant tissues only accounting for a small proportion (<1%). However, direct grazing from snails can be significant for macrophytes in clear‐water mesocosms with low epiphyton, as previously documented (Cao, Zhang, Sun, & Li, 2019). Nevertheless, earlier studies have been relatively short or conducted in microcosms (e.g., petri dishes) (Cao et al., 2014; Li, Liu, & Gu, 2009; Xiong et al., 2008), which do not necessarily reflect the interactions observed among snails, epiphyton, and macrophytes in real lakes with low epiphyton biomass.

Here, we conducted a long‐term experiment in an outdoor clear‐water mesocosm system to investigate the effects of herbivorous snails on macrophyte growth under different snail densities and plant structures when epiphyton biomass is low. We hypothesized that in a clear‐water system, (a) high snail density would significantly inhibit the growth of submerged macrophytes compared with low snail density and (b) submerged macrophytes with distinct structures would show different responses to snail grazing.

## 2 MATERIALS AND METHODS

### 2.1 Experimental material

Anhui Province has one of the densest distributions of freshwater shallow lakes in China, containing some 64 natural lakes with areas larger than 100 hm^2^. In the current study, we collected four submerged macrophyte species, that is, *Myriophyllum spicatum*, *Potamogeton wrightii*, *P. crispus*, and *P. oxyphyllus*, from a shallow stream (30°42′48″N, 116°49′06″E) in Anhui Province, China. The species were precultivated separately in plastic tanks (diameter: 56 cm, height: 100 cm) filled with untreated river water for one month prior to the experiment. The four species are common submerged macrophytes in this area, and all possess long stems that extend to the surface, but differ in leaf morphology and phenology: that is, *M. spicatum* has highly incised leaves; *P. oxyphyllus* has narrow, undissected leaves; and both *P. wrightii* and *P. crispus* have flat and undissected leaves, with the latter exhibiting early growth and reproduction as an adaptation to colder temperatures in subtropical climate zones. In addition, compared with *M. spicatum*, the stems of *P. oxyphyllus*, *P. wrightii*, and *P. crispus* are thinner; furthermore, *P. oxyphyllus* has only one vascular bundle in its stem, whereas the others have multiple vascular bundles (Editorial Committee of Flora of China, 2013).

*Radix swinhoei* is a pulmonata freshwater snail, widely distributed throughout Asia, including Japan, Thailand, India, Burma, and China, and found in diverse habitats, including lakes, ponds, streams, rivers, and rice fields (Liu, Zhang, & Wang, 1979). As a generalist plant grazer and detritus feeder, *R. swinhoei* feeds extensively on submerged macrophytes (Li, Liu, Hu, & Yang, 2009; Xiong et al., 2008). In the current study, *R. swinhoei* snails were harvested in Wuhan Botanical Garden (30°33′01″N, 114°25′48″E, Hubei Province, China) and precultivated in tanks (1 × 1 × 1 m^3^) filled with tap water at a field station in Anhui Province, China (30°42′48″N, 116°49′06″E). The snails were maintained without food for 48 hr prior to the start of the experiment (Li, Liu, Hu, et al., 2009).

### 2.2 Experimental design

Twenty‐four plastic tanks (diameter: 56 cm, height: 100 cm) were used in the experiment: that is, four macrophyte species (MC) × two snail densities (SN) × three replicates.

#### 2.2.1 Treatment 1: Macrophyte species

For the four macrophyte species (*M. spicatum* (Ms), *P. oxyphyllus* (Po), *P. wrightii* (Pw), and *P. crispus* (Pc)), top shoots with the same initial biomass (ca. 1.5 g fresh weight) were planted into plastic pots (top diameter: 29 cm, bottom diameter: 20 cm, height: 18 cm) filled two‐thirds with soil presoaked in river water for one week. There were four individuals of each species in each pot, which were then randomly placed into one tank (diameter: 56 cm, height: 100 cm) filled with untreated river water. An outlet tap was installed on the tank at a height of 50 cm, and one half of the water in the tank was manually discharged and replaced with municipal water each month after each water sampling. The water retention time was two months. A net‐covered overflow hole was added to the tank at a height of 95 cm to ensure the correct water level under natural precipitation and to avoid snail escape.

#### 2.2.2 Treatment 2: Snail densities

To mimic more realistic conditions, we established two snail density treatments: that is, low snail density (LS) and high snail density (HS). When the experiment started (12 May 2016), five similar‐sized snails (average fresh weight ca. 0.4 g per individual) were added to the mesocosm for HS treatment, with no snails added for LS treatment. During the HS experiment, three snails with the same total weight (ca. 1.5 g fresh weight) were added to the mesocosm every month after water sampling; in addition, artificial removal of new snails (which probably hatched from sediment collected from natural rivers or from eggs attached to the macrophytes) was conducted for LS treatment. The HS density was based on previous study (Cao et al., 2014; Li, Liu, Hu, et al., 2009).

### 2.3 Sampling schedule and measurement of water physiochemistry, plant traits, and snail biomass

The experiment lasted from May 2016 to April 2017. After one month precultivation, water samples were collected to determine initial total nitrogen (TN), total phosphorus (TP), and phytoplankton chlorophyll *a* (Chl*a*) content before SN treatment. During the experiment, sampling was conducted in June, August, September, November, December, February, and April. During each sampling event, dissolved oxygen (DO) and light attenuation coefficients (Kd) were first determined. DO was measured using a ProODO Optical Dissolved Oxygen Instrument (YSI, USA) at a depth of 30 cm, and Kd was calculated by light attenuation at a depth of 30 cm using the formula in Kirk (1977) based on a Li‐192 underwater quantum sensor and data logger Li‐1400 (LI‐COR, USA). The outlet taps were then opened, and water samples were collected in 250‐ml and 1‐L plastic bottles. The 1‐L water samples were used for phytoplankton Chl*a* content, which was determined by ethanol extraction after filtering through Whatman GF/C filters (Huang, Chen, & Cai, 1999). The 250‐ml water samples were taken back to the laboratory for the determination of TN, TP, pH, and alkalinity. Both TN and TP contents were ascertained using spectrophotometry after digestion with K_2_S_2_O_8_ solution (Huang et al., 1999), and alkalinity was determined by titration using 0.1 mmol/L HCl. Finally, plant traits, including plant height, plant volume inhabited (PVI), ramet number, and flower number (if the plants flowered), were measured and recorded. PVI was calculated by (average coverage) × (community plant height)/water depth.

At the end of the experiment, macrophyte and epiphyton samples were collected. The second mature leaves (for Pc and Pw) and new 10‐cm long shoots (for Po and Ms) were harvested and stored in plastic bags at 4°C for the determination of epiphyton biomass (Cao et al., 2014). The remaining macrophytes were harvested and cleaned in tap water, together with the plants collected for epiphyton, and then dried at 80°C for 48 hr to determine above‐ and belowground biomass. The ratio of belowground and aboveground biomass (BG/AG) was calculated. The water in the plastic tanks was then discharged, and snails in the mesocosm were collected by a 500‐μm invertebrate net. Snail biomass and abundance were recorded.

### 2.4 Statistical analyses

For the data collected before the SN treatments in May, two‐way analysis of variance (ANOVA) was used to analyze the initial conditions, with MC and SN as the two main factors. The water physiochemistry and plant trait data (including plant height, PVI, and ramet number) collected during the experiment were analyzed by repeated‐measures ANOVA (RM‐ANOVA), followed by the Student–Newman–Keuls (S‐N‐K) post hoc test. Data were log (*x* + 1)‐transformed when needed to satisfy the assumptions for Mauchly's test of sphericity; otherwise, the Greenhouse–Geisser value was used. Statistical results are shown in Table 1. When the interaction between MC and SN was significant, the data were split into subdatasets for each macrophyte species. Student's *t* test was used to analyze differences in SN treatments for each macrophyte species.

**TABLE 1:** Statistical significance tests on changes in macrophyte indicators and water chemistry in MC and SN treatments, as determined by repeated‐measures ANOVA

Indicator	Treatment			Time	Treatment × Time			S‐N‐K test for treatment	
	MC	SN	MC × SN		MC × Time	SN × Time	MC × SN ×Time	MC	SN
Plant height	253.73, ***	0.69, NS	9.45, ***	88.25, ***	18.39, ***	0.37, NS	1.52, NS	Pc, Po < Pw < Ms	
Ramet number	32.01, ***	4.72, *	0.27, NS	568.87, ***	80.37, ***	2.15, NS	2.19, **	Pc < Ms, Pw < Po	HS < LS
PVI	160.02, ***	8.32, *	5.02, *	14.44, ***	6.19, ***	0.31, NS	1.33, NS	Pc < Pw, Po < Ms	HS < LS
Phytoplankton Chl*a*	0.45, NS	0.17, NS	0.64, NS	21.07, ***	0.61, NS	1.03, NS	0.90, NS		
TN	3.00, NS	0.47, NS	0.18, NS	35.80, ***	0.94, NS	1.46, NS	1.55, NS		
TP	2.21, NS	2.34, NS	1.10, NS	44.30, ***	2.37, **	1.31, NS	1.53, NS		
Alkalinity	11.24, ***	0.01, NS	2.86, NS	104.86, ***	8.26, ***	1.50, NS	2.72, **	Po < Pc, Ms, Pw	
Kd	16.17, ***	1.96, NS	3.04, NS	53.67, ***	3.03, ***	0.54, NS	0.60, NS	Po < Pc, Pw < Ms	
pH	1.38, NS	0.03, NS	0.53, NS	147.13, ***	1.32, NS	1.24, NS	1.08, NS		
DO	4.22, *	0.77, NS	2.41, NS	235.27, ***	2.16, *	0.66, NS	0.79, NS	Pw < Ms, Pc, Po	

Note

MC, SN, and Time refer to three factors: macrophyte species treatments (Pw, Pc, Ms, and Po), snail density treatments (HS and LS), and sampling time, respectively. Values are *F*; significant *p‐*values (NS = not significant, *p < .05, **p < .01, ***p < .001).

For plant biomass, snail biomass and abundance, flower number, and epiphyton biomass data collected at the end of the experiment, two‐way ANOVA (Table 2) was used to explore the effects of MC and SN treatments, followed by the S‐N‐K post hoc test. We used SPSS 24.0 for statistical analyses. All data are presented as mean ± *SD*.

**TABLE 2:** Statistical significance tests on changes in macrophyte indicators, epiphyton, and snail biomass in MC and SN treatments, as determined by two‐way ANOVA

Indicator	Treatment			S‐N‐K test for treatment	
	MC	SN	MC × SN	MC	SN
Total biomass	43.16, ***	6.03, *	1.19, NS	Pw < Pc <Ms, Po	HS < LS
AG biomass	101.45, ***	8.46, *	1.17, NS	Pw < Pc <Ms < Po	HS < LS
BG biomass	7.30, **	2.37, NS	0.84, NS	Pw, Po, Pc < Ms	
BG/AG biomass	65.01, ***	0.84, NS	0.88, NS	Po < Pc, Ms < Pw	
Flower number	1.93, NS	0.51, NS	0.35, NS		
Epiphyton	0.97, NS	0.67, NS	1.22, NS		
Snail biomass	6.43, **	21.93, ***	0.24, NS	Po < Pw	LS < HS
Snail abundance	4.12, *	6.08, *	1.19, NS	Po, Pc < Pw	LS < HS

Note

BG and AG refer to below‐ and aboveground biomass, respectively. MC and SN refer to two factors: macrophyte species treatments (Pw, Pc, Ms, and Po) and snail density treatments (HS and LS), respectively. Values are *F*; significant *p‐*values (NS = not significant, *p < .05, **p < .01, ***p < .001).

## 3 RESULTS

### 3.1 Water physiochemistry and epiphyton biomass

Prior to SN treatment, no significant differences were found in TN, TP, and phytoplankton Chl*a* content (two‐way ANOVA, *F* < 2.74, *p* > .05 for both treatments; see Table S1). After one month preculture, alkalinity in the Pc treatment was significantly lower than that in the other three MC treatments (alkalinity among MC treatments, *F* = 12.90, *p* < .001), and pH was slightly higher in the Ms treatment (8.77 ± 0.06) than that in Pw (8.58 ± 0.07), Po (8.58 ± 0.10), or Pc (8.57 ± 0.05) (pH among MC treatments, *F* = 8.11, *p* < .01).

**FIGURE 1:**
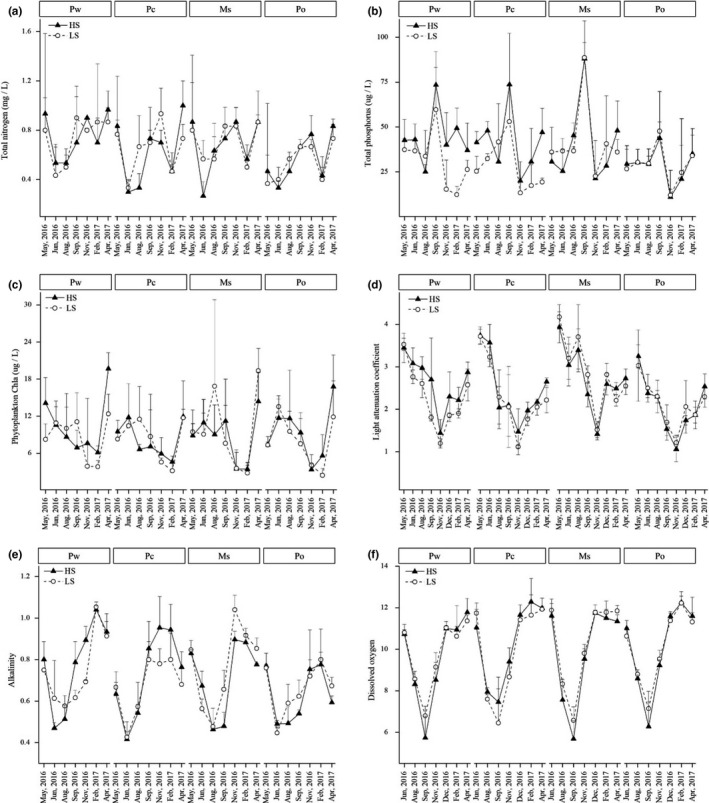
Changes in water physiochemistry at different measurement dates under two snail densities (HS and LS) with four macrophyte species (Pw, Pc, Ms, and Po). (a) Total nitrogen; (b) total phosphorus; (c) phytoplankton Chl*a*; (d) light attenuation coefficient; (e) alkalinity; and (f) dissolved oxygen. Values are displayed as mean ± standard error of at least three replicates

**FIGURE 2:**
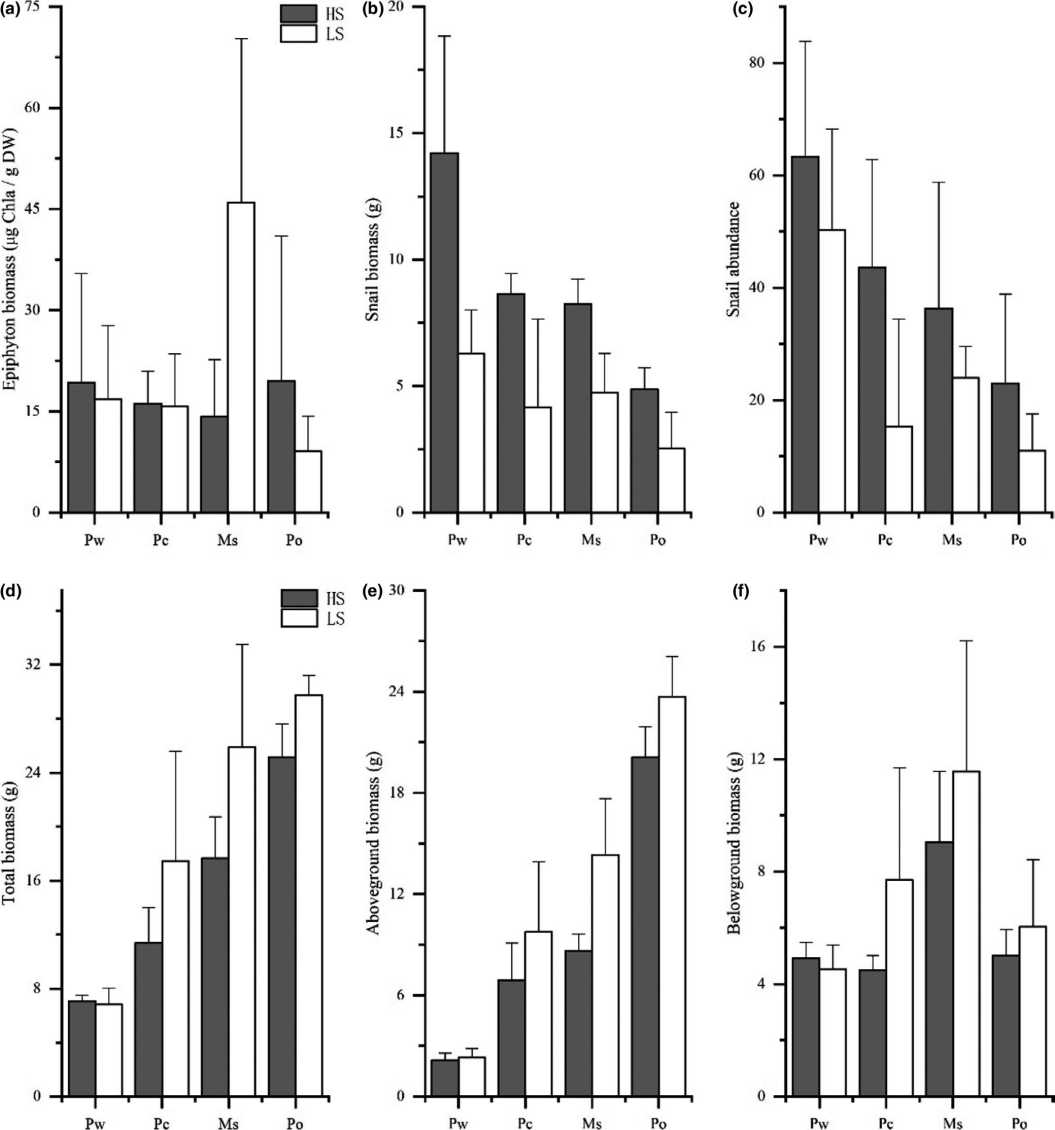
Variation in biometric parameters in response to snail densities (HS and LS) in four macrophyte species (Pw, Pc, Ms, and Po). (a) Epiphyton; (b) snail biomass; (c) snail abundance; (d) total biomass; (e) aboveground biomass; and (f) belowground biomass. Values are displayed as mean ± standard error of at least three replicates

Results also showed that TN, TP, phytoplankton Chl*a*, and pH did not differ among the four MC or two SN treatments during the whole experiment (Figure 1a–c; Table 1). Relatively low concentrations of TN (0.66 ± 0.27 mg/L) and TP (36 ± 20 μg/L) and a phytoplankton Chl*a* range of 14.8 ± 4.9 μg/L were recorded (see Table S1). Similarly, epiphyton biomass was low (19.6 ± 15.9 μg Chl*a* per g dry weight (DW)), and there were no significant differences between MC and SN treatments (Figure 2a; Table 2). In addition, Kd, alkalinity, and DO did not differ between the two SN treatments; however, for the four MC treatments, Kd and alkalinity were lowest in Po (2.10 ± 0.65 and 0.65 ± 0.14, respectively) and DO was lowest in Pw (9.78 ± 1.89 mg/L) (Figure 1d–f; Table 1).

### 3.2 Snail biomass and abundance

In the HS treatment, both snail biomass and abundance (36.5 ± 16.5 g/m^2^ and 169 ± 92 ind/m^2^, respectively) were twice as high as that under LS treatment (18.0 ± 9.6 g/m^2^ and 102 ± 80 ind/m^2^, respectively) (Figure 2b,c; Table 2). For the MC treatments, snail biomass and abundance were both higher in Pw than in Po (Table 2; see Figure S1).

### 3.3 Indicators of submerged macrophytes

Two‐way ANOVA showed that the total biomass and aboveground biomass of the macrophytes were higher under LS treatment (Table 2), with no significant difference between HS and LS treatments found for belowground biomass. Total biomass and aboveground biomass were lowest in Pw and highest in Po, and belowground biomass was highest in Ms (Figure 2d–f; Table 2).

**FIGURE 3:**
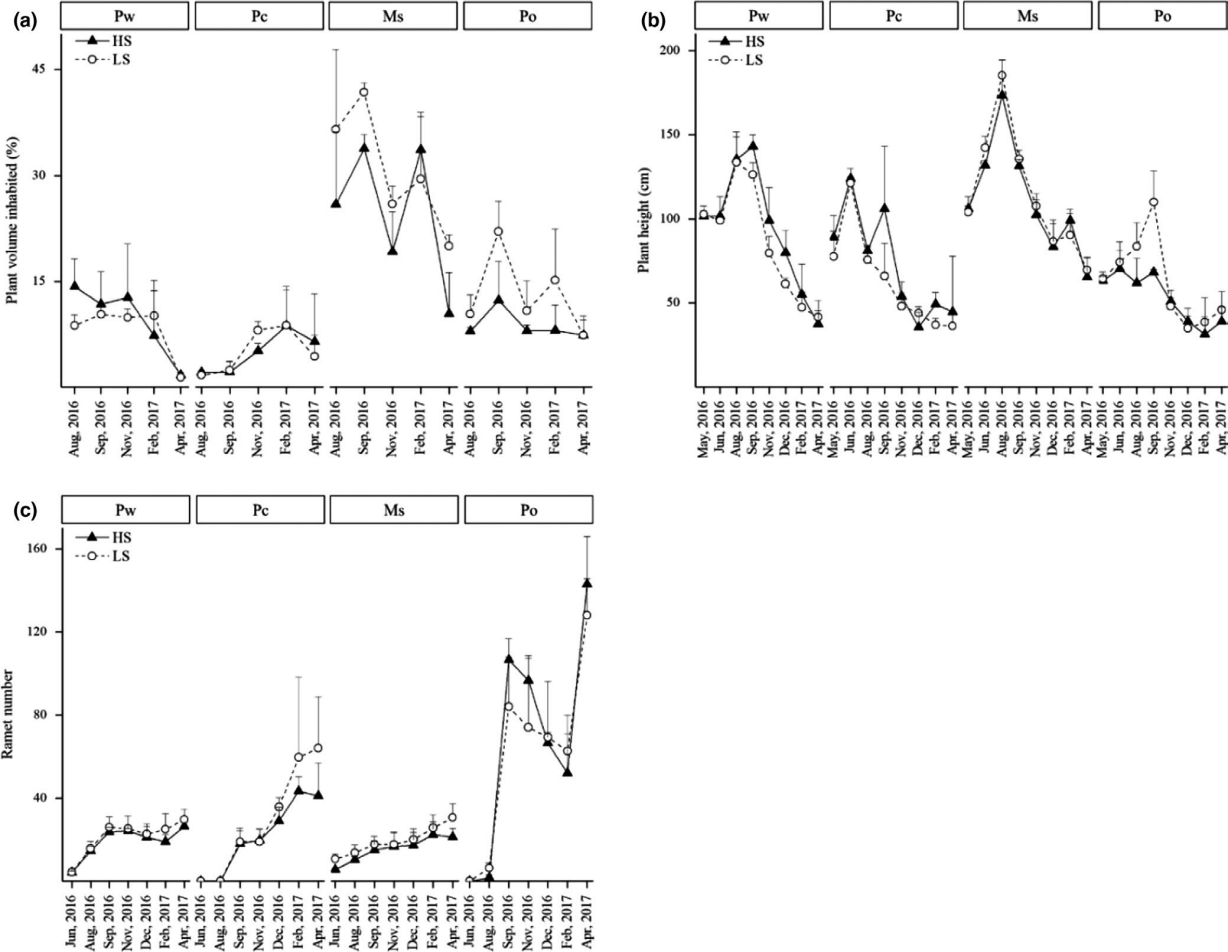
Changes in plant volume inhabited (a), plant height (b), and ramet number (c) at different measurement dates under two snail densities (HS and LS) with four macrophyte species (Pw, Pc, Ms, and Po). Values are displayed as mean ± standard error of at least three replicates

Results also showed that the PVI of submerged macrophytes was lower in HS treatment than in LS treatment (Figure 3a; Table 1). However, plant height and PVI showed significant interactions among the MC and SN treatments, and thus, further analysis was conducted for each species. Results indicated that the height of Po and the PVI of Ms were both higher under LS treatment than those under HS treatment (Figure 3a,b; see Table S2).

According to the RM‐ANOVA results, plants produced more ramets under LS treatment than HS treatment (Table 1), especially in June and August (Figure 3c). Flower number did not differ among the four species or between the two SN treatments (Table 2; see Figure S2).

Plant height, PVI, and ramet number differed significantly among the different species (Table 1). Ms had the greatest plant height and PVI, whereas Pc had the lowest plant height, PVI, and ramet number (Figure 3a–c).

## 4 DISCUSSION

Our results indicated that high snail density reduced total biomass of submerged macrophytes and ramet number compared with low snail density, thus supporting our first hypothesis. In addition, plant height and PVI showed species‐specific responses to snail density, partially supporting our second hypothesis.

The mesocosms in this study were under relatively clear‐water conditions, with low phytoplankton biomass. Previous studies have shown that epiphyton biomass is usually less than 200 μg Chl*a* per g DW in low‐nutrient freshwater mesocosms and lakes (Cao et al., 2017; Romo, Villena, & García‐Murcia, 2007). In this experiment, the epiphyton biomass was relatively low (19.6 ± 15.9 μg Chl*a* per g DW). Thus, it was assumed that the growth of macrophytes was not limited by light availability (Phillips et al., 1978). Alkalinity was also relatively low, but all chosen macrophytes exhibit strong bicarbonate usage ability (Yin, Li, Madsen, Maberly, & Bowes, 2017). Thus, the limitation of carbon supply was considered negligible in our study. Though TN and TP in the water column were low during the experiment, the nutrient levels did not differ significantly between the two snail density treatments. Thus, potential nutrient limitations were not considered important factors for differences in macrophyte growth under the two different snail densities.

The naturally occurring density of snails found in Yangtze lakes is 18.05 ± 7.43 g/m^2^ (on average), with strong seasonal change (Wang, Pan, Liang, & Wang, 2006). This rate is comparable to that found under LS density treatment (18.0 ± 9.6 g/m^2^, 102 ± 80 ind/m^2^) by the end of the experiment. While our results agree well with previous studies (Elger & Lemoine, 2005; Li, Liu, Hu, et al., 2009; Sheldon, 1987), showing that snails graze directly on living macrophytes, we also found some differences. For example, Li, Liu, and Gu (2009) reported that the growth of submerged macrophyte *Vallisneria natans* (wrongly named *V. spiralis* in the reference) is suppressed by high snail density (i.e., 240 ind/m^2^) but promoted by low snail density (80 ind/m^2^), with no positive or negative effects observed by snail grazing at 160 ind/m^2^. In contrast, we observed the same negative effects of high snail density at 170 ind/m^2^. These different responses of submerged macrophytes to snails could be ascribed to epiphyton biomass on the macrophytes and nutrient levels. In Li, Liu, and Gu (2009), the mesocosm was under eutrophic conditions (TN > 2.6 mg/L, TP > 70 μg/L in the present snail treatments) and high epiphyton biomass (20–30 μg/cm^2^) (Cao et al., 2014; Köhler, Hachoł, & Hilt, 2010; Shurin, Clasen Jessica, Greig Hamish, Pavel, & Thompson Patrick, 2012). Snails generally prefer to graze on epiphyton, rather than macrophytes (Cao et al., 2019; Li, Liu, Hu, et al., 2009). However, given the low epiphyton biomass in our study, direct grazing by snails on submerged macrophytes was expected to be high. Our results showed that aboveground biomass, but not belowground biomass, of macrophytes was lower under high snail density treatment, consistent with Xiong et al. (2008), suggesting that snails can inhibit plant growth by feeding on the leaves of submerged macrophytes or damaging the soft stems of certain species.

Nevertheless, macrophyte species respond differently to snail grazing (Cao et al., 2014; Li, Liu, Hu, et al., 2009; Underwood et al., 1992). *Myriophyllum spicatum* has a strong allelopathic ability for antiherbivory by increasing the production of secondary chemicals (e.g., phenols) when being eaten (Le Bagousse‐Pinguet, Liancourt, Gross, & Straile, 2012). In contrast to its grazing‐tolerant features, the PVI of *M. spicatum* in the current study was more sensitive to high snail density than was found for the other macrophyte species. This may reflect an antiherbivory avoidance mechanism, whereby fewer aboveground parts (i.e., leaves and stems) are produced for snails to graze upon (Anderson & Briske, 1995). Although snail biomass and abundance were both lower on *P. oxyphyllus* than on the other three species, *P. oxyphyllus* height was the only parameter showing a difference among snail density treatments, indicating the high sensitivity of this species to snail grazing. Compared with the other plants, *P. oxyphyllus* has thinner stems (Editorial Committee of Flora of China, 2013) and is thus more likely to be damaged by snails.

Snails are an important part of the food chain and often used as the basal line for isotopic analysis of the aquatic food web (Post, 2002). The role of snails in regulating macrophyte communities relies on the nutrient levels of lakes and the biomass of epiphyton (Cao et al., 2017; Underwood et al., 1992; Yang et al., 2020). Our clear‐water mesocosm experiment indicated that snails at high density can graze on and reduce the growth of submerged macrophytes under low epiphyton biomass conditions. However, real lakes have much more complex food webs, and the effects of different snail densities may change under such ecosystems due to the existence of vertebrate species such as fish. For example, in shallow lakes, predatory fish can feed on snails and thus inhibit their effects through top‐down control.

In summary, compared with low snail density, the inhibitory effects of long‐term high snail density on submerged macrophytes by direct feeding may be greater than the positive effects resulting from epiphyton clearance when under clear‐water conditions with low epiphyton biomass. This is of great significance for the management of clear‐water lakes. For example, the manual addition of invertebrates (i.e., snails) into such lakes could be a practical way in which to regulate the growth and community composition of submerged macrophytes in shallow lake ecosystems when the impact from predatory fish is weak. As the present results were obtained via a mesocosm study, additional studies in real lakes are warranted to verify the above conclusions.

## CONFLICT OF INTEREST

None declared.

## AUTHOR CONTRIBUTION

**Yongwei Zhi:** Data curation (equal); Formal analysis (equal); Writing‐original draft (equal). **Yang Liu:** Investigation (supporting); Methodology (equal); Writing‐original draft (supporting). **Wei Li:** Conceptualization (lead); Project administration (equal); Supervision (equal); Writing‐review & editing (equal). **Yu Cao:** Conceptualization (equal); Funding acquisition (lead); Investigation (equal); Supervision (equal); Writing‐original draft (equal); Writing‐review & editing (equal).

## Supporting information

Supplementary Material

## Data Availability

The datasets generated during the current study are available in the Dryad repository (https://doi.org/10.5061/dryad.j6q573n9z).
