# Supplementary material for: Responses of four submerged macrophytes to freshwater snail density (Radix swinhoei) under clear‐water conditions: A mesocosm study
Source: Ecol Evol. 2020 Jun 23;10(14):7644–53. doi: 10.1002/ece3.6489 (PMC7391322; doi:10.1002/ece3.6489)

**Supplementary Material**

**Table S1:** Changes in water physiochemistry and chlorophyll-a at different measurement dates in mesocosm.

| Indicator | May 16 | | Jun. 16 | | Aug. 16 | | Sep. 16 | | Nov. 16 | | Dec. 16 | | Feb. 17 | | Apr. 17 | |
| --- | --- | --- | --- | --- | --- | --- | --- | --- | --- | --- | --- | --- | --- | --- | --- | --- |
|  | Mean | SD | Mean | SD | Mean | SD | Mean | SD | Mean | SD | Mean | SD | Mean | SD | Mean | SD |
| Chla (ug/L) | 9.1 | ± 2.9 | 11.1 | ± 3.1 | 10.5 | ± 6.4 | 8.7 | ± 4.7 | 4.6 | ± 3.2 |  |  | 4.0 | ± 1.9 | 14.8 | ± 4.9 |
| TN (mg/L) | 0.73 | ± 0.41 | 0.40 | ± 0.15 | 0.53 | ± 0.17 | 0.74 | ± 0.19 | 0.81 | ± 0.13 |  |  | 0.55 | ± 0.23 | 0.86 | ± 0.15 |
| TP (ug/L) | 33.1 | ± 10.1 | 35.3 | ± 9.5 | 34.0 | ± 11.1 | 66.0 | ± 23.6 | 19.5 | ± 14.2 |  |  | 28.0 | ± 19.3 | 35.3 | ± 13.4 |
| Alk | 0.76 | ± 0.08 | 0.51 | ± 0.11 | 0.53 | ± 0.08 | 0.67 | ± 0.14 | 0.84 | ± 0.14 |  |  | 0.90 | ± 0.13 | 0.77 | ± 0.13 |
| pH | 8.62 | ± 0.10 | 8.88 | ± 0.27 | 7.54 | ± 0.04 | 8.40 | ± 0.53 | 8.54 | ± 0.25 | 8.40 | ± 0.42 | 7.32 | ± 0.17 | 9.42 | ± 0.21 |
| Do |  |  | 11.2 | ± 0.64 | 8.2 | ± 0.56 | 6.5 | ± 1.10 | 9.2 | ± 0.65 | 11.5 | ± 0.38 | 11.7 | ± 0.87 | 11.6 | ± 0.49 |
| Temp | 29.6 | ± 1.0 | 29.3 | ± 0.4 | 30.3 | ± 0.2 | 25.2 | ± 0.2 | 13.9 | ± 0.2 |  |  | 9.3 | ± 0.2 | 10.8 | ± 0.2 |

**Table S2:** Statistical significance tests on changes in plant height and PVI in SN treatments for each submerged macrophyte, as determined by one-way ANOVA. MC refers to macrophyte species (Pw, Pc, Ms and Po). SN refers to snail density treatments (HS and LS). Values are F; significant *p-*values (NS = not significant, * = *p* < 0.05, ** = *p* < 0.01, *** = *p* < 0.001).

| Indicator | MC | Treatment | SNK test for treatment |
| --- | --- | --- | --- |
|  |  | SN | SN |
| Plant height | Ms | 1.97, NS |  |
|  | Po | 35.43, *** | HS < LS |
|  | Pc | 3.13, NS |  |
|  | Pw | 5.89, NS |  |
| PVI | Ms | 9.61, * | HS < LS |
|  | Po | 6.57, NS |  |
|  | Pc | 0.01, NS |  |
|  | Pw | 1.40, NS |  |

**Figure S1:** Changes in snail biomass and abundance under four macrophyte species (Pw, Pc, Ms, and Po) scenarios.


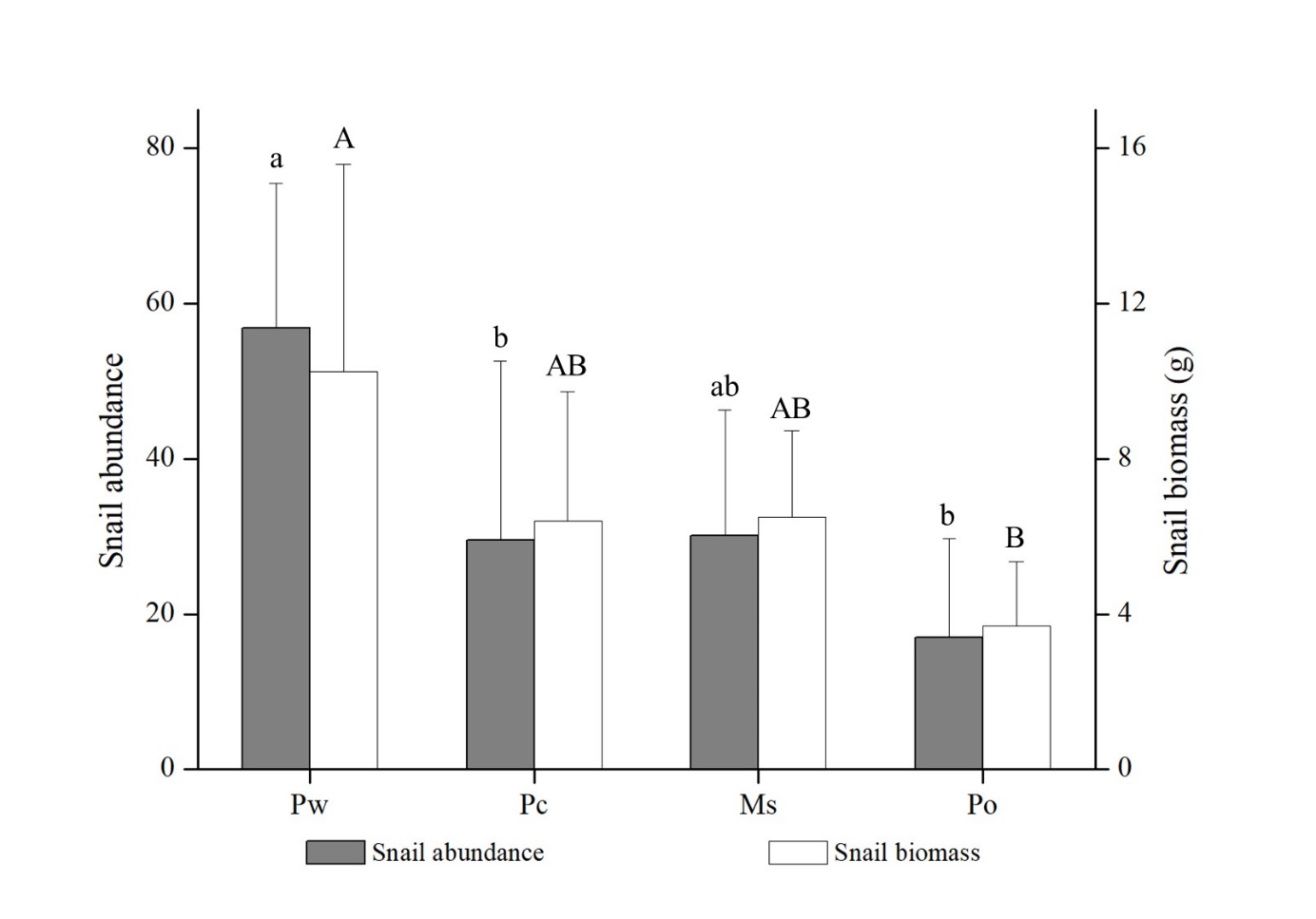


**Figure S2:** Flower number and belowground/aboveground biomass in response to snail densities (HS and LS) in four macrophyte species (Pw, Pc, Ms, and Po).


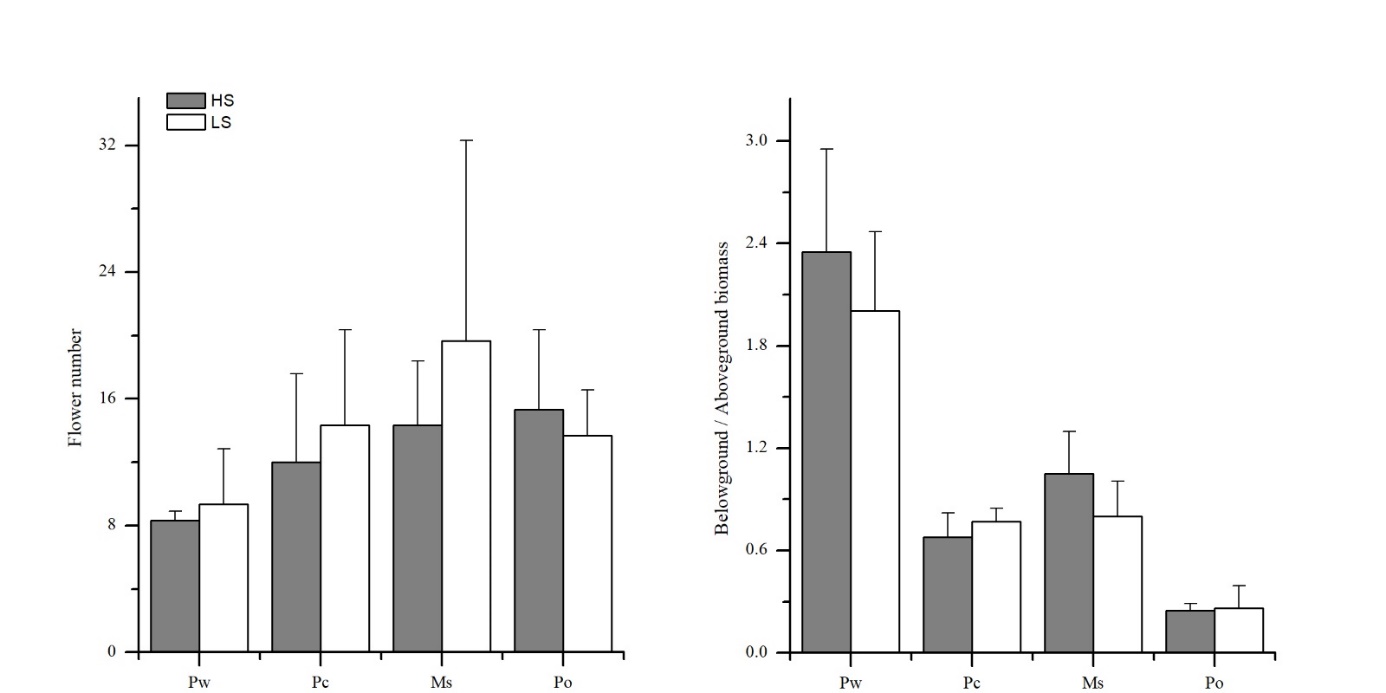

Supplement: Supplementary file 1 — Supplementary Material [file ECE3-10-7644-s001.docx]
